# Gastrointestinal angiodysplasias diagnosed using video capsule endoscopy in 15 dogs

**DOI:** 10.1111/jvim.16677

**Published:** 2023-03-03

**Authors:** Alice Defarges, Jenny Stiller, Jeffrey A. Solomon

**Affiliations:** ^1^ University of Guelph Ontario Veterinary College Guelph Ontario Canada; ^2^ Universität Leipzig Veterinärmedizinische Fakultät Klinik Leipzig Germany; ^3^ Infiniti Medical, LLC Palo Alto California USA

**Keywords:** anemia, gastrointestinal bleeding, hematochezia, hemolytic anemia, melena, microcytosis, occult, overt

## Abstract

**Background:**

Angiodysplasia (AGD) is rarely diagnosed in dogs with gastrointestinal bleeding (GIB) and is reported in case reports in dogs.

**Objective:**

Describe signalment, clinical and diagnostic features of dogs with gastrointestinal (GI) AGD diagnosed by video capsule endoscopy (VCE).

**Animals:**

Dogs with overt or suspected GIB which underwent VCE.

**Methods:**

Dogs for which a VCE was submitted for overt or suspected GIB from 2016 to 2021 were selected retrospectively. Medical records and full‐length VCE recordings where AGDs were initially detected, were reviewed by 2 trained internists. AGD was considered definitive if 2 readers detected it. Signalment, clinical signs, blood work, medications, concurrent diseases, findings of previous conventional endoscopy, and surgical exploration (if applicable) of dogs with AGD were recorded.

**Results:**

Definitive AGD was diagnosed in 15 of 291 (5%) dogs (12 males, 3 females). Twelve (80%) had overt GIB, 11 (73%) had hematochezia, and 6 (40%) had microcytic and hypochromic anemia. AGD was missed by conventional endoscopy in 9/9 dogs and exploratory surgery in 3/3 dogs. Thirteen capsules were administered by mouth (1 incomplete study), and 2 via endoscopy directly into the duodenum. AGD was visualized in the stomach of 3 dogs, in the small intestine of 4, and in the colon of 13 dogs.

**Conclusion and Clinical Importance:**

Although rare, AGD should be considered in dogs with suspected GIB after a negative conventional endoscopy or surgical exporation. Video capsuel endoscopy appears to be a sensitive test to identify AGD within the GI tract.

**Abbreviations**
AGD: angiodysplasia
CKD: chronic kidney disease
GI: gastrointestinal
GIB: gastrointestinal bleeding
IBD: inflammatory bowel disease
IMHA: immune‐mediated hemolytic anemia
VCE: video capsule endoscopy

## 1 INTRODUCTION

Gastrointestinal (GI) vascular malformations, referred to as angiodysplasia (AGD) or vascular ectasia, are aberrations in the typical architecture of blood vessels; arterial, venous, and capillary vessels can be affected.^1^ Disruption of these fragile dilated mucosal vessels induces bleeding. AGD can affect any segment of the GI tract. In humans, AGD are most commonly found in the small bowel.^2^, ^3^ The exact etiology of the disease in humans is unknown. Clinical signs can vary from subclinical without any visible bleeding (occult GI bleeding) to visible hemorrhage (overt GI bleeding) in the form of hematemesis, hematochezia, or melena.^4^, ^5^ The GI hemorrhage can become chronic and intermittent resulting in iron deficiency anemia.

AGD in humans is recognized as an important cause of lower gastrointestinal bleeding (GIB) in elderly adults^6^ but also occurs in younger patients.^7^

The diagnosis of AGD in humans is often delayed because of the intermittent nature of the bleeding and the lack of sensitivity of the abdominal ultrasound, conventional endoscopy in cases of small bowel AGD, and surgical exploration to detect abnormal vessels.^8^ The diagnosis is made based on results of endoscopic visualization of the gastrointestinal mucosa. Histopathological confirmation is not required.^1^ Video capsule endoscopy (VCE) has many advantages over conventional endoscopy, as it is a less expensive, outpatient procedure with the further advantage of being able to evaluate the entire GI tract using a higher magnification. Therefore, VCE has become the diagnostic test of choice to detect AGD in humans.^9^, ^10^, ^11^

Only 7 cases of GI vascular anomalies are reported in dogs, and all abnormalities were located in the rectum or colon.^12^, ^13^, ^14^, ^15^, ^16^, ^17^ This suggests that these disorders might be less common or underdiagnosed in dogs compared with people.^12^, ^13^, ^14^, ^15^, ^16^, ^17^ The median duration of clinical signs is 33 months (range: 0.25‐60 months),^13^, ^14^, ^15^, ^16^, ^17^, ^18^ which illustrates that making a diagnosis of AGD is challenging. Three of 7 dogs underwent surgical exploration that did not identify the presence of AGD.^12^, ^14^, ^15^ The AGD diagnosis was made with conventional endoscopy in all 7 cases. However, considering that in people AGD is most commonly diagnosed in the small intestine (SI), it is possible that AGD is missed in some dogs that only receive bidirectional endoscopy without further assessment of the complete small intestine.

The use of VCE has become more popular in dogs as a minimally invasive tool to detect GIB.^19^, ^20^, ^21^, ^22^, ^23^ This procedure is well tolerated in dogs and yields a diagnosis in over 75% of dogs with overt GIB.^19^ Because repeating VCE is less expensive and invasive than conventional endoscopy and surgical exploration, it is also a promising test for intermittent GIB.

The objective of this retrospective study was to report the signalment, clinical and diagnostic features of AGD diagnosed by VCE in a large cohort of dogs with suspected or overt GIB.

## 2 MATERIALS AND METHODS

Reports of dogs with overt (within 2 weeks of capsule administration) or suspected (unexplained anemia) GIB were retrieved from a large database of VCE studies (ALICAM, Infiniti Medical, LLC, Redwood City, CA) submitted for diagnostic evaluation to [Infiniti Medical, LLC, Redwood City, CA] between November 1, 2016 and March 1, 2021. If a dog had been diagnosed initially with AGD by VCE, full‐length recordings were independently reviewed by 2 trained investigators: reader 1(AD, nonblinded to history, signalment and initial diagnosis) and reader 2 (JSt, blinded to history, signalment and initial diagnosis) to confirm the diagnosis. Reader 1 and 2 had read more than 200 and 58 VCE studies respectively before this study. Esophageal, gastric, and small intestinal transit times were recorded. If the capsule reached recording capacity while still in the stomach, the study was considered “incomplete.” If the capsule was delivered endoscopically directly into the small intestine or if it turned off while still in the SI, the study was considered “partial.”

A lesion of AGD was defined by the presence of a clearly demarcated, bright‐red, flat lesion, consisting of tortuous and clustered capillary dilatations within the mucosal layer.^24^ The number of frames where AGD lesions were visible was recorded as follows: 1 frame, 2 to 5 frames, 6 to 10 frames or more than 10 frames. If both investigators (reader 1 and 2) observed a lesion of AGD in ≥2 frames, it was considered definitive. If only 1 reader observed a lesion of AGD on ≥2 frames, it was considered suspicious. For each dog, the presence of a AGD lesion was recorded along with the location (stomach, SI or colon) as well as the probability of bleeding based on a previously reported score.^25^ The location of lesions within the small intestine observed by VCE was defined by percentage % of total small bowel transit time (first third, second third and last third). The following data were recorded for each dog: signalment (age, breed, sex, weight), geographical location, clinical signs (type and duration), the reason for the VCE exam (unexplained anemia, overt GIB or a combination of both), complete blood count and biochemistry results, resting/post‐ACTH stimulation cortisol concentrations, TLI, folate, cobalamin concentrations, iron panel, abdominal ultrasound, conventional endoscopy (upper ± lower), GI biopsies histopathology, exploratory laparotomy, historical and concurrent diseases, and medications.

Descriptive statistics were performed for all recorded data. Fisher's exact test was used to determine if there was a statistically significant difference in clinical signs between dogs diagnosed with AGD compared to those without. For purposes of comparison, dogs with suspicious lesions (interpreted as AGD by 1 reader) were grouped as not having AGD. Significance level *α* was set .05. All statistical analyses were performed using SAS/SAT 9.4 (SAS Institute Inc 2013. Cary, NC: SAS Institute Inc).

## 3 RESULTS

From November 1, 2016 to March 1, 2021, a total of 322 dogs underwent a VCE. Thirty‐one were excluded for the following reasons: lack of complete blood count, lack of medical records including history, lack of anemia or overt GIB. Two hundred ninety‐one dogs underwent VCE for suspected or overt GIB: 81 dogs had unexplained anemia, and 210 dogs had overt GIB. Eight board‐certified internists were involved in the initial readings of the 291 capsules. Eighteen of 291 studies were reviewed by AD and JSt as AGD was diagnosed or suspected on the initial reports (done by 4 readers including AD for 8 cases). Of these, AGD was confirmed (definitive) in 15 of 291 (5%) dogs. In the remaining 3 dogs, AGD lesions were suspected by 1 reader but not by the other. Among the 210 dogs with overt GIB who were administered a capsule, 12 (6%) were diagnosed with definitive AGD.

### 3.1 Signalment of the 15 dogs with definitive GI AGD lesions

Of the 15 dogs with AGD, 5 were intact males, 7 were neutered males, and 3 were spayed females. Their median age was 108 months (range: 8‐152 months). Fourteen dogs were older than 6 years. The median weight of the dogs was 14 kg (range: 4.5‐49.5 kg). Nine dogs were living in the USA, 3 in Canada, 2 in the United Kingdom, and 1 in Australia. The Schnauzer (3 miniature, 1 giant) was the most represented breed, followed by terrier mix breed (4) and 1 each of the following: golden retriever, basset hound, Cavalier King Charles, Border collie, shih tzu, Norwegian Elkhound, and toy poodle.

### 3.2 Clinical signs of the 15 dogs with definitive AGD lesions

Clinical signs of dogs diagnosed with AGD compared to those without are presented in Table 1. Twelve of 15 dogs (80%) with AGD had overt GIB (hematochezia, melena, or a combination of both). None had hematemesis. Three of 15 dogs (20%) with AGD diagnosed via VCE had occult GIB.

**TABLE 1:** Number of dogs with suspected or overt GIB, hematochezia, melena, hematemesis, and a combination of hematochezia and melena, among the 15 dogs with definitive angiodysplasia and among the 276 dogs without definitive angiodysplasia respectively.

	Number (and %) of dogs with definitive AGD on VCE	Number of dogs (and %) without confirmed AGD on VCE	*P* value
Overt GI bleeding	12 (80)	198 (72)	.7677
Suspected GI bleeding	3 (20)	78 (28)	.7677
Hematochezia	11 (73)	65 (24)	.0001
Melena	8 (53)	133 (48)	.7945
Hematemesis	0 (0)	44 (16)	.1385
Hematochezia and melena	8 (53)	32 (12)	.0002

*Note*: Angiodysplasia was diagnosed by VCE by 2 readers independently. If only 1 reader diagnosed AGD, it was considered a suspicious lesion, and the dog was classified as not having AGD.

Abbreviations: AGD, angiodysplasia; GI, gastrointestinal; GIB, gastrointestinal bleeding; NA, not applicable; VCE, video capsule endoscopy.

Aside from signs of GIB, other clinical signs were lethargy (8), diarrhea (8), weight loss (5), decreased appetite (3), pica (3), tenesmus (1), and vomiting (1). The median time from clinical onset to diagnosis of AGD was 10 weeks (range: 3 days to 1 year). Eleven of 76 dogs (14%) with hematochezia had AGD. Eight of 40 dogs (20%) with hematochezia and melena had AGD. Hematochezia and hematochezia combined with melena were significantly more common in the group of dogs with GIB and AGD compared to the group of dogs with GIB without AGD (*P* = .0001 and *P* = .0002, respectively). The other variables were not significantly different between the 2 groups (Table 1).

### 3.3 Concurrent diseases of the 15 dogs with definitive GI AGD lesions

Three dogs had historical chronic kidney disease, 7 had hepatobiliary disease (vacuolar hepatopathy, cholecystitis, microhepatica), while others had recurrent aspiration pneumonia (1), arteriovenous fistula of the shoulder (1), mitral valve disease (3), hypothyroidism (1), pulmonary hypertension (1), previous splenectomy (4), bladder urolithiasis (2), and intervertebral disc disease (1). One dog had a history of immune‐mediated thrombocytopenia which was resolved the previous year.

Nine dogs were fed a hypoallergenic diet for a suspected (4) or confirmed (5) inflammatory bowel disease by histopathology.

Based on the diagnostic algorithm for immune‐mediated hemolytic anemia (IMHA),^26^ testing was supportive of IMHA in 1 dog based on the presence of spherocytes and autoagglutination on the blood smear^26^ and suspicious for IMHA in 9 dogs. Direct agglutination tests were performed on 3 dogs and were negative.

### 3.4 Ongoing treatment of the 15 dogs with definitive GI AGD lesions

Eight dogs had been treated with prednisone (median: 2 mg/kg/day; range: 1‐4 mg/kg/day) for a median of 3.25 months (range: 2 weeks to 5 months) for suspected IMHA. In 2 dogs, the prednisone was discontinued 2 weeks before the capsule was administered. At the time of capsule administration, 5 dogs were on immunosuppressive dose of prednisone. Two of the dogs had been on budesonide, 1 for 1 month and 1 for 6 months (0.14 mg/kg q24h and 0.1 mg/kg q24h respectively). Fifteen dogs were receiving proton pump inhibitors, and 15 were receiving sucralfate. One dog was administered maropitant, and Vitamin B12. One was treated with barium, and another 1 with Yunnan Baiyao. One dog was administered pimobendan, and 1 was administered iron supplementation. Eight dogs received a median of 3 transfusions (range 1‐8) before the VCE over a median of 30 days (range 1‐55).

### 3.5 Clinicopathological data in dogs with definitive GI AGD lesions

All dogs had a complete blood count and 14 had a biochemistry profile performed within 1 week before the VCE. All 15 dogs were anemic. The median hematocrit was 15% (range: 9‐37%). The anemia was regenerative in 6 dogs, poorly regenerative in 9 dogs, normocytic and normochromic in 2 dogs, microcytic and hypochromic in 6 dogs, and macrocytic and hypochromic in 7 dogs.

Serum iron concentration, total iron binding capacity, iron saturation, unsaturated iron binding capacity, and ferritin concentrations were measured in 4 dogs. The results of the iron panel indicated the presence of iron deficiency anemia in all dogs.^27^ Histopathologic evaluation of bone marrow core performed in 2 dogs revealed the absence of stainable iron, which was consistent with iron deficiency anemia.

Eight dogs underwent a prothrombin time test and a partial thromboplastin time test; all were normal.

### 3.6 Conventional imaging studies in dogs with definitive GI AGD lesions

Abdominal ultrasound (AUS) showed no relevant findings in 7/12 dogs who had a US performed. It revealed 2 gastric foreign bodies in 1 dog, thickened intestinal layers (2), microhepatica and coarse echotexture of the liver (1), peritoneal fluid (4), increased prostatic size (2), splenomegaly (1), 1 hypoechoic nodule in the caudal pole of the left adrenal gland (1), and splenic infarcts (1). The intestines could not be evaluated in 1 dog because of a moderate volume of ingesta.

A total of 9 dogs had conventional endoscopy performed. The endoscopy was performed a median of 2.5 weeks (1‐24) before (7), or at the same time as, capsule administration (2). One dog underwent only upper GI endoscopy, while upper and lower GI endoscopies were performed in 8 dogs. Endoscopic biopsies were collected and histopathology showed gastric mural fibrosis in 1 dog, helicobacter‐like organisms in the stomach of 2 dogs, lymphoplasmocytic enteritis in 4 dogs, mild lymphangiectasia in 1 dog, mild lymphoplasmocytic and eosinophilic enteritis in 1 dog, and colitis in 2 dogs (eosinophilic, and lymphoplasmocytic respectively). In another dog, abnormally high intermediate lymphocytes in intestinal biopsies led to the performance of a PCR for antigen receptor rearrangements. Based on the polyclonal results, lymphoma was considered unlikely. One dog had an abdominal CT scan with angiography that did not reveal any relevant findings in the abdomen.

### 3.7 Exploratory laparotomy in dogs with definitive GI AGD

Exploratory laparotomy was performed in 3 dogs, 1 at 8 days, 1 at 10 days, and 1 at 14 days before the capsule was administered. In 1 dog, the liver and the spleen were enlarged; a splenectomy was performed. Histopathology analysis of the spleen revealed extramedullary hematopoiesis. The GI tract appeared normal and palpated normally, so no GI biopsies were sampled. In 2 dogs, no relevant macroscopic lesions were observed during surgery. Surgical biopsies of the GI tract revealed histologically normal stomach, small intestine, and colon.

### 3.8 Video capsule endoscopy of the 15 dogs with definitive GI AGD

The capsule was administered orally in 13 dogs and directly into the duodenum by endoscopy in 2 dogs (Table 2). Fifteen capsules were excreted in the feces. It took a median of 24 hours (range: 8‐48 hours) for the capsules to be excreted in 10 dogs. In 5 dogs, the time of excretion was not reported.

**TABLE 2:** Method of administration of the video capsule endoscopy (oral or endoscopic), completeness of each study (complete/incomplete/partial), and location of the angiodysplasia (stomach, small intestine, or colon) for each of the 15 dogs with definitive AGD.

Dog	Delivery method	Completeness (C/I/P)	Location of AGD		
			Stomach	SI	Colon
1	Endoscopic	P	NE	+	+
2	Oral	C	−	−	+
3	Oral	C	+	−	−
4	Oral	I	+	NE	NE
5	Oral	C	−	+	+
6	Oral	C	−	+	+
7	Endoscopic	P	NE	+	+
8	Oral	C	−	−	+
9	Oral	C	−	−	+
10	Oral	C	−	−	+
11	Oral	C	+	−	+
12	Oral	C	−	−	+
13	Oral	C	−	−	+
14	Oral	C	−	−	+
15	Oral	C	−	−	+

*Note*: If the capsule reached recording capacity while still in the stomach, the study was considered “incomplete.” If the capsule was delivered endoscopically directly into the small intestine or if it turned off while still in the SI, the study was considered “partial.”

Abbreviations: AGD, angiodysplasia; C, complete; I, incomplete (incomplete only stomach evaluated); NE, not evaluated; P, partial (only intestines evaluated); SI, small intestine.

The median capsule transit time was 8 seconds (range: 5‐57 seconds) in the esophagus. One of 13 capsules (7%) administered by mouth reached recording capacity before reaching the small intestine. The capsule evaluated the stomach in 13 dogs, and the small intestine and colon in 14 dogs (Table 2). The median gastric and intestinal capsule transit times were 103 minutes (range: 6‐487 minutes) and 132 minutes (range: 38‐304 minutes), respectively, for complete studies (12). The gastric mucosa looked irregular in 11 dogs (cobblestone [9], polypoid [1], and mosaic [1] appearance).

**FIGURE 1:**
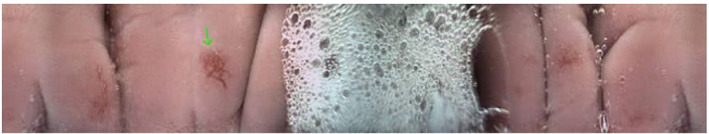
Recorded VCE frame showing a panoramic view of gastric mucosa of a 11‐year‐old male castrated Miniature Schnauzer (9 kg) showing abnormal vessels (vascular ectasia) in the stomach.

**FIGURE 2:**
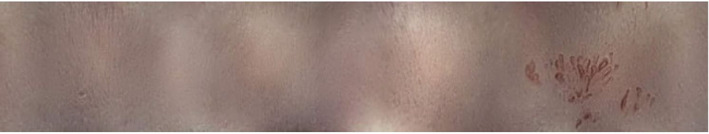
Recorded VCE frame showing a panoramic view of duodenal mucosa of a 11‐year‐old male castrated Miniature Schnauzer (9 kg) showing abnormal vessels (vascular ectasia) in the duodenum (same dog as Figure 1).

**FIGURE 3:**
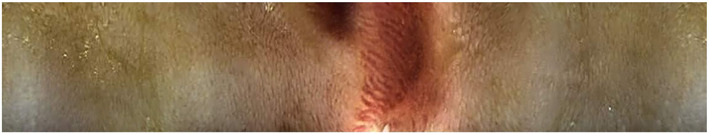
Recorded VCE frame showing a panoramic view of the ileal mucosa of a 10‐year‐old female spayed Terrier Mix (10 kg) showing abnormal vessels (vascular ectasia) in the ileum.

**FIGURE 4:**
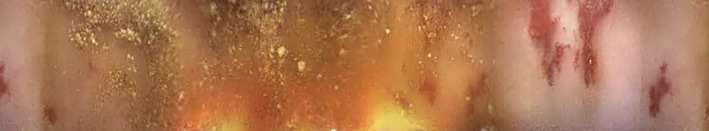
Recorded VCE frame showing a panoramic view of the colonic mucosa of a 11‐year‐old male castrated Mini Schnauzer (9 kg) showing abnormal vessels (vascular ectasia) in the colon (same dog as Figures 1 and 2).

**FIGURE 5:**
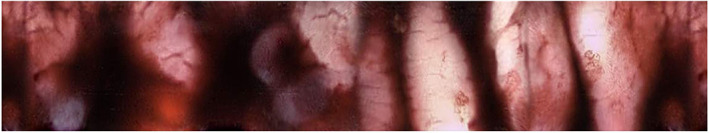
Recorded VCE frame showing a panoramic view of colonic mucosa of a 11‐year‐old male castrated Golden Retriever (33 kg) showing abnormal vessels (vascular ectasia) in the colon and substantial bleeding.

Reader 1 and 2 detected AGD in more than 10 frames in 14 dogs, and in 5 to 10 frames in 1 dog. Angiodysplasias were observed in the stomach in 3 dogs (Figure 1). One of these studies was incomplete. One out of 3 had colonic AGD as well (Table 2). AGD was visualized in the first third of the small intestine in 1 dog (Figure 2), in the distal third of the small intestine in 3 dogs (Figure 3), and in the colon in 13 dogs (Figures 4 and 5). Eleven of 12 dogs with complete VCE studies had colonic AGD. The 2 dogs with capsules administered endoscopically had colonic AGD (Table 2).

Recent bleeding was observed in the intestines (2 dogs), and in the colon (2 dogs) along with AGD lesions. Active bleeding was observed in the stomach (6 dogs), in the intestine (2 dogs), and in the colon (8 dogs) along with AGD lesions. No ulcerative or erosive lesions were observed in any of the 15 dogs.

### 3.9 VCE of the 276 dogs not diagnosed with definitive AGD lesions

The capsule was administered orally in 265 dogs and endoscopically in 11 dogs. Among them, 170 (62%) studies were complete, 97 of 276 (35%) were incomplete, and 9 of 276 (3%) were partial (delivered into the duodenum).

In 3 dogs, whose initial report indicated the presence of AGD, readers 1 and 2 disagreed. One reader reported AGD while the other 1 reported gastric erosions (Dog 1 and 2). In Dog 3, reader 1 detected AGD lesions in ˃5 frames per lesion, while reader 2 detected several AGD lesions in the colon, each on 1 frame. The presence of active bleeding in the colon impaired the visualization of the mucosa. Dog 3 had ulcerative colitis diagnosed on endoscopic biopsies.

## 4 DISCUSSION

This retrospective study reports 15 cases of GI AGD diagnosed by VCE in dogs. AGD was not detected during previous surgical explorations in 3/3 dogs and by conventional endoscopy in 9/9 dogs. This study suggests that VCE might be more sensitive than conventional endoscopy and surgery to detect GI AGD in dogs.

Five percent of dogs which had a VCE for suspected or overt GIB had AGD. This number is comparable to humans, where 2% to 8% of GIB are secondary to AGD.^28^ In humans, the frequency of AGD in the colon being a cause of lower intestinal bleeding is 40% among adults,^28^ but the frequency is unknown for children and for dogs.

The most common clinical signs among the 15 dogs with definitive AGD were hematochezia, lethargy, melena and diarrhea. This is consistent with reports of disease in dogs.^12^, ^13^, ^14^, ^15^, ^16^ Dogs might collapse^13^ which could be associated with sudden bleeding in the GI tract. In this study looking at dogs undergoing VCE, 11 out of 76 dogs (14%) with GIB and hematochezia, had AGD and up to 20% of dogs with GIB and concurrent melena and hematochezia that underwent VCE had AGD. Hematochezia and hematochezia combined with melena were significantly more common in the group of dogs with GIB and AGD compared to the group without AGD. The results were not adjusted for comparison to avoid underpowering the tests and increasing type II error rates.^29^ These findings suggest that AGD should be considered in adult dogs with GIB and concurrent hematochezia or hematochezia and melena.

In this study, diagnosis occurred a median of 10 weeks after the start of the clinical signs or unexplained anemia. Similarly, in humans a delay in diagnosis of 2 months to 3 years has been reported.^8^, ^30^ The delay in diagnosing AGD in dogs is likely because of the intermittent characteristic of the GIB, and the possible lack of sensitivity of the conventional endoscopy and surgical exploration. Additionally, bleeding lesions may be identified on traditional endoscopy but misinterpreted as erosive or ulcerative lesions instead of abnormal vessels.

AGD cannot be identified using an AUS; however, it can, in some patients, be observed by conventional endoscopy. In the veterinary literature, AGD was diagnosed through colonoscopy in 7 dogs.^14^, ^15^ However, AGD should not be ruled out based on the absence of abnormal vessels on conventional endoscopy because intermittent bleeding, localization of AGD lesions within the mid SI, lower magnification (versus VCE), and overinflation of the bowel with air can cause lesions to blanche and be invisible. Furthermore, the AGD can easily be confused with iatrogenic minor mucosa trauma from the endoscope. The appearance of the lesions can also be influenced by the patient's blood pressure, degree of anemia, and effective blood volume, as well as the routine use of opioids^31^ that are vasoconstrictive.^32^

In humans, AGD is rarely detected during surgical exploration because the lesions are not always visible on the serosal surface of the GI.^30^ Similarly, in 2 veterinary case reports and in 3 dogs in our study, surgical exploration failed to detect AGD.^14^, ^15^ Moreover, in humans, AGD can be missed during histopathologic observation of GI biopsies. Most lesions are not diagnosed histologically because of their small size (shrinkage with processing of the specimen, or error in targeting the biopsy).^33^ Similarly, in our study, surgical biopsies of the colon did not reveal AGD in dogs with confirmed AGD on endoscopy.

In humans, VCE is the preferred method of small‐bowel evaluation after inconclusive conventional endoscopy because of higher or at least equivalent diagnostic performance as compared with other, more invasive techniques, such as push enteroscopy, mesenteric angiography, computed tomography and intraoperative enteroscopy.^10^, ^34^, ^35^ VCE is a noninvasive procedure with a high rate of complete small bowel exploration (>90%) and high diagnostic performance (>60%) used to diagnose GIB lesions in humans.^10^, ^11^ Compared to conventional endoscopy, VCE facilitates visualization because of the higher magnification, the evaluation of the entire GI tract, and the absence of insufflation, sedation, or general anesthesia that can mask AGD. Besides, because of its lower cost and lack of invasiveness, it can be repeated easily. In 3 dogs in our study, the capsules identified AGD in the distal small intestine that could not previously be reached by a conventional endoscope.

VCE has several limitations. Lesion localization is estimated according to the transit time and the use of the pylorus and cecum as landmarks, but these lack precision^36^ and can be problematic if surgical resection is indicated. Nonetheless, VCE can help in deciding the best insertion route for endoscopy if endoscopic cauterization is considered (upper or lower).

In humans, 1 study compared capsule endoscopy and intraoperative enteroscopy in 47 patients with obscure GIB.^37^ The sensitivity and specificity of VCE were 95% and 75%, respectively. The positive predictive value of VCE was 95%, and the negative predictive value was 86%.^37^ The sensitivity and specificity of VCE to detect AGD is still unknown in people and in dogs.

The ability of VCE to detect a true AGD is affected by the difficulty in distinguishing an abnormal vessel (AGD) from a submucosal hemorrhage, a red spot, or an erosive lesion. Because of the random movement of the capsule, lesions are sometimes present in only 1 frame and reorientation of the capsule is not possible to confirm the type of lesion. However, based on the clinical history and assessment of the lesions using criteria from the human literature,^7^, ^24^ the reader can determine that other diagnoses are less likely. In our study, reader 1 and 2 disagreed on gastric lesions in 2 dogs. It is likely easier to distinguish true AGD from other lesions in tubular organs (such as intestine or colon) versus in the stomach where the capsule is further away from the mucosa. In that case, the presence of AGD in other GI segments would support the presence of AGD in the stomach. In this study, readers 1 and 2 disagreed on interpretation of colonic lesions in Dog 3. The reader who was blinded to the history, could see suspicious lesions but only on 1 frame because of the blood pooling in the colon. The ulcerative lesions have been misinterpreted as AGD. It is uncertain if ulcerative colitis and AGD can coexist.

The ability of VCE to detect AGD is affected by the bowel preparation, the transit time, and the time between the bleeding episode and the test. VCE studies that are performed soon after GIB have higher diagnostic yields than later studies in humans. The European guidelines therefore recommend performing VCE within 14 days of a bleeding event.^38^ The examination is limited to the number of frames on which the lesion is visible. Because of the strong duodenal peristalsis, the lack of insufflation and motion control, lesions can be missed in the proximal small intestine.^37^, ^39^ Additionally, lesions can be missed if the capsule reaches recording capacity while in the stomach. Nevertheless, considering the low price and minimal invasiveness of VCE, a repeat study is reasonable after prokinetic administration or endoscopic delivery directly into the duodenum.

In our study, 35% VCE recordings of the 276 dogs without definitive AGD were incomplete, which is comparable to the rate of 13% to 47% of incomplete studies published so far in the veterinary literature.^19^, ^21^, ^22^ Consequently, AGD could have been missed in some of these dogs. In humans, several risk factors, such as GIB, have been associated with incomplete studies.^40^ A high‐fat diet, chronic enteropathy, and recent use of opioids may increase the risk of incomplete studies in dogs.^19^ The use of prokinetics might be beneficial.^41^

Biopsies cannot be performed by the device, which limits the diagnostic ability of VCE. However, considering the limited sensitivity of histopathology for AGD, biopsies are not essential. Finally, this procedure lacks therapeutic abilities in contrast to conventional endoscopy.^13^

Conventional endoscopy should still be considered the first test to perform in a dog with ongoing GIB.^42^ However, VCE is useful in dogs with persistent obscure GIB with negative gastroscopy, colonoscopy, or surgical exploration results or for dogs whose bleeding has stopped spontaneously (inactive GIB) and urgent hemostasis is not required.

AGD was present in the colon in 11 dogs with complete VCE studies and 2 dogs with capsules administered endoscopically. One out of 3 dogs with definitive gastric AGD had incomplete studies, so colonic lesions may have been missed. The higher prevalence of AGD in the colon is comparable with older human studies when capsule endoscopy was not used.^35^, ^43^ Recent studies evaluating the use of capsule endoscopy showed that the majority of AGD are detected in the small intestine.^2^, ^3^

In our study, 2/12 dogs (17%) with complete studies had AGD in multiple GI segments (stomach, small intestine, and colon). In comparison, in humans, approximately 60% of adult patients had lesions in more than 1 location in the GI tract.^2^ However, multifocal AGD may have been underestimated in our study because some VCE exams were incomplete or partial. It is still uncertain if the presence of multifocal AGD is associated with a more advanced disease in humans or in dogs.

Our study reports gastric AGD in dogs. Two of 3 dogs with definitive gastric AGD were older than 8 years. In humans, gastric and small intestinal AGD are more common than colonic AGD in young patients with a rare genetic vascular disorder called “hereditary hemorrhage telangiectasia” (HHT), also known as Osler‐Weber‐Rendu syndrome.^44^, ^45^ No cases of suspected HHT are reported in the veterinary literature.

In our study, 8/15 dogs with definitive AGD and 1/3 dogs with suspicious AGD had been treated with high doses of steroids for ≥2 weeks for a suspected IMHA. As none of the dogs fulfilled the diagnostic criteria for IMHA,^26^ the anemia may have been secondary to chronic GIB. Among 7 canine AGD reports, 4 dogs had received steroids.^13^, ^15^, ^17^ Interestingly, in humans, long‐term anabolic steroid therapy induced bleeding angiectasia.^46^ Because administration of prednisone or hyperadrenocorticism can result in hypercoagulability and thrombotic diseases,^47^, ^48^, ^49^, ^50^, ^51^, ^52^, ^53^ high doses of glucocorticoid could have induced the formation of microthrombi in the GI vasculature, leading to obstruction and AGD formation. Unfortunately, none of our dogs were tested for hypercoagulability, so this hypothesis cannot be verified.

Our study emphasizes the importance of considering GIB as a possibility when some features of IMHA are not present, such as hyperbilirubinemia, presence of spherocytosis, positive direct agglutination test. Similarly, the absence of microcytosis or hypochromasia should not rule out GIB, as these changes can take several weeks to develop.^4^ Evaluation of erythrocyte cytograms provided by automated hematology analyzer and measurement of reticulocyte indices can provide early indicators of iron deficiency anemia.^54^

The presence of inflammatory bowel disease in 5 dogs in our study and 1 dog in a previous report^16^ suggests a potential association between AGD and IBD in dogs.

Aortic stenosis,^55^, ^56^ liver disease, CKD,^35^ parasitism,^57^ coagulopathies,^1^, ^58^ and Von Willebrand disease^59^ are known risk factors for AGD in humans. Considering the small number of cases in dogs, it is not possible to yet determine risk factors for AGD among dogs.

The majority of the dogs in our study were older than 6 years. This is comparable with previous reports in the veterinary literature where 4/7 dogs were older than 8 years^12^, ^13^, ^15^, ^17^ and only 2 were less than 1 year of age.^14^, ^16^ Similarly, AGD is more common in human patients aged >60 years.^6^ The etiology of AGD is unknown, but 1 potential age‐related explanation is a partial obstruction of submucosal veins leading to a weakening of the precapillary sphincters, creating congestion.^60^ In humans, it has been suggested that chronic obstruction of the submucosal veins leads to capillary dilatation and eventually to the failure of a precapillary sphincter.^1^, ^30^, ^61^

In our study, the majority of dogs diagnosed with definitive AGD were males (12/15, 80%). Similarly, 6/7 cases reported in the veterinary literature were male.^12^, ^13^, ^14^, ^15^, ^16^, ^17^ A statistical sex predilection could not be examined because of the small number of dogs in our study, nor has 1 been documented in humans.^62^

In our study, 3 of 15 dogs with definitive AGD were Miniature Schnauzers born ≥6 years prior and living in North America. The dogs were not related but a breed predisposition is possible. No other Schnauzers were previously reported.^12^, ^13^, ^14^, ^17^ Further studies are needed to determine if some breeds are predisposed to AGD.

This study has several limitations. First, as per its retrospective nature, the management of the cases was not standardized, and some data may have been missed in the medical records. Second, it is possible that the interpretation of VCE findings by reader 1 was biased because this reader was not blinded to signalment, clinical history and initial VCE findings. Therefore, a lesion was only considered as definitive AGD if both readers were in agreement as reader 2 was blinded to the dog's information and also to the selection criterion for the VCE studies.

Third, the selection of VCE studies that were reviewed by the 2 readers (AD, JSt) was based on the initial diagnosis made on the first reading. Therefore, it is possible that AGD lesions were missed in the 276 videos that were not reviewed. However, since the initial readings were performed by experienced board‐certified internists, the risk of missed lesions is considered low.

It was not possible to review the medical records of the 273 dogs who underwent VCE for suspected or overt GIB during the same period of time and for which no AGD was detected initially. For this reason, no solid conclusion can be offered regarding a possible correlation of breed, IMHA, or IBD and GI AGD.

## CONFLICT OF INTEREST STATEMENT

Dr. Alice Defarges has been a consultant for Infiniti Medical since March 2020; Dr. Jeff Solomon is affiliated with Infiniti Medical, LLC, Palo Alto, California; Dr. Jenny Stiller has been a consultant with Infiniti Medical since January 2022. The other author declares no conflict of interest.

## OFF‐LABEL ANTIMICROBIAL DECLARATION

The authors declare no off‐label use of antimicrobials.

## INSTITUTIONAL ANIMAL CARE AND USE COMMITTEE (IACUC) OR OTHER APPROVAL DECLARATION

The authors declare no IACUC or other approval was needed.

## HUMAN ETHICS APPROVAL DECLARATION

The authors declare human ethics approval was not needed for this study.

## Supporting information


**Data S1.** Supporting Information
