# Supplementary material for: Gastrointestinal angiodysplasias diagnosed using video capsule endoscopy in 15 dogs
Source: J Vet Intern Med. 2023 Mar 3;37(2):428–36. doi: 10.1111/jvim.16677 (PMC10061205; doi:10.1111/jvim.16677)
Supplement: Supplementary file 1 — Data S1. Supporting Information [file JVIM-37-428-s001.pdf]

In the material and Methods:

Line 81:

[1: Pale red spots; 2: Bright red spots; 3: Recent bleeding (clot, and digested blood debris); 4: Active bleeding (fresh blood)]

In the results (subheading "*Clinicopathological data in dogs with definitive GI AGD lesions*

")

Line 162

Two patients had hypoalbuminemia (2 and 2.4 g/dL) based on their respective laboratory reference ranges. Alanine transaminase and alkaline phosphatase were increased (≥2 times the upper end of the reference range) in 3 and 4 dogs, respectively. All of these patients had been on prednisone for more than 2 weeks.

Line 173

Four dogs had a GI panel performed including trypsin-like immunoreactivity and folate and cobalamin concentrations; all results were normal. One dog had a serum resting cortisol test performed, and the result was higher than 2 μg/dl.

Table 2: Method of administration of the video capsule endoscopy (oral or endoscopic), completeness of each study (complete/incomplete/partial), and location of the angiodysplasia (stomach, small intestine, or colon) for each of the 15 dogs with definitive AGD.

| Dog | Delivery method | Completeness (C/I/P) | Location of AGD | | |
|---|---|---|---|---|---|
| | | | Stomach | SI | Colon |
| 1 | Endoscopic | P | NE | + | + |
| 2 | Oral | C | - | - | + |
| 3 | Oral | C | + | - | - |
| 4 | Oral | I | + | NE | NE |
| 5 | Oral | C | - | + | + |
| 6 | Oral | C | - | + | + |
| 7 | Endoscopic | P | NE | + | + |
| 8 | Oral | C | - | - | + |
| 9 | Oral | C | - | - | + |
| 10 | Oral | C | - | - | + |
| 11 | Oral | C | + | - | + |
| 12 | Oral | C | - | - | + |
| 13 | Oral | C | - | - | + |
| 14 | Oral | C | - | - | + |
| 15 | Oral | C | - | - | + |

Note: If the capsule reached recording capacity while still in the stomach, the study was considered "incomplete." If the capsule was delivered endoscopically directly into the small intestine or if it turned off while still in the SI, the study was considered "partial."

AGD: angiodysplasia; C: Complete; I: Incomplete (incomplete only stomach evaluated), P: Partial (only intestines evaluated); NE: not evaluated; SI: Small Intestine
